# Sex and Genetic Factors Determine Osteoblastic Differentiation Potential of Murine Bone Marrow Stromal Cells

**DOI:** 10.1371/journal.pone.0086757

**Published:** 2014-01-28

**Authors:** Stefano Zanotti, Ivo Kalajzic, Hector Leonardo Aguila, Ernesto Canalis

**Affiliations:** 1 Department of Research, Saint Francis Hospital and Medical Center, Hartford, Connecticut, United States of America; 2 University of Connecticut School of Medicine, University of Connecticut Health Center, Farmington, Connecticut, United States of America; 3 Department of Reconstructive Sciences, University of Connecticut Health Center, Farmington, Connecticut, United States of America; 4 Department of Immunology, University of Connecticut Health Center, Farmington, Connecticut, United States of America; INSERM U1059/LBTO, Université Jean Monnet, France

## Abstract

Sex and genetic factors determine skeletal mass, and we tested whether bone histomorphometric parameters were sexually dimorphic in femurs from 1 to 6 month old C57BL/6 mice. Trabecular bone volume declined more rapidly in female mice than in male littermates because of enhanced bone resorption. Although bone formation was not different between sexes, female mice exhibited a higher number of osteoblasts than male littermates, suggesting that osteoblasts from female mice may have a reduced ability to form bone. To determine the impact of sex on osteoblastogenesis, we investigated the potential for osteoblastic differentiation of bone marrow stromal cells from C57BL/6, Friend leukemia virus-B (FVB), C3H/HeJ and BALB/c mice of both sexes. Bone marrow stromal cells from female FVB, C57BL/6 and C3H/HeJ mice exhibited lower *Alpl* and *Osteocalcin* expression and alkaline phosphatase activity, and formed fewer mineralized nodules than cells from male littermates. Proliferative capacity was greater in cells from male than female C57BL/6, but not FVB, mice. Sorting of bone marrow stromal cells from mice expressing an α-Smooth muscle actin-green fluorescent protein transgene, revealed a higher yield of mesenchymal stem cells in cultures from male mice than in those from female littermates. Sex had a modest impact on osteoblastic differentiation of mesenchymal stem cells. To determine the influence of sex and genetic factors on osteoblast function, calvarial osteoblasts were harvested from C57BL/6, FVB, C3H/HeJ and BALB/c mice. *Alpl* expression and activity were lower in osteoblasts from C57BL/6 and C3H/HeJ, but not FVB or BALB/c, female mice than in cells from littermates. Sex had no effect on osteoclastogenesis of bone marrow cultures of C57BL/6 mice, but osteoblasts from female mice exhibited higher *Rankl* and lower *Opg* expression than cells from male littermates. In conclusion, osteoblastogenesis is sexually dimorphic and influenced by genetic factors.

## Introduction

Human and rodent males attain higher peak bone mass during growth than females [1]. Trabecular bone declines more rapidly and at a younger age in maturing female than in male C57BL/6 mice, so that adult female mice have less cancellous bone than male mice [2]. Whereas sex differences in skeletal mass and structure are evident, the mechanisms involved are understood poorly. Sex hormones regulate bone remodeling, but skeletal differences related to gender become evident prior to the onset of sexual maturity [3], [4]. Furthermore, genetic determinants regulate bone mass acquisition, and differences in androgen and estrogen levels do not seem to account for skeletal sexual dimorphism [5], [6].

The balance of osteoblast and osteoclast activity maintains skeletal remodeling and integrity, and alterations in osteoclast or osteoblast number or function can lead to changes in bone mass [7]. Osteoblast number is determined by the replication and differentiation of bone marrow mesenchymal stem cells toward osteoblasts, the death of mature cells and their differentiation into lining cells or osteocytes [8], [9]. Expression of the myxovirus resistance 1 promoter, or of the mesenchymal gene marker α-Smooth muscle actin (*αSma*) defines a population of osteoblast progenitors able to differentiate into osteoblasts, both *in vitro* and *in vivo* [10]–[12]. Multinucleated osteoclasts derive from the fusion of mononuclear cell precursors of the hematopoietic lineage residing in the bone marrow. Osteoclast precursors express receptor activator of nuclear factor-κb (Rank), and activation of Rank following contact with cells expressing Rank-ligand (Rankl), is required for osteoclastogenesis. Rankl activity is inhibited by the soluble Rankl receptor osteoprotegerin (Opg), so that the Rankl to Opg ratio determines the number and activity of osteoclasts [13], [14].

In this study, we explored the mechanisms determining sexual dimorphism in bone mass by performing histomorphometric analysis of cancellous bone from femurs of male and female C57BL/6 littermate mice. To investigate whether sex and genetic factors have an impact on osteoblast differentiation and function, we studied the potential for osteoblastogenesis of bone marrow stromal cells and the function of calvarial osteoblasts from male or female C57BL/6, tropism for Friend leukemia virus-B (FVB), BALB/c or C3H/HeJ mice. In addition, we tested whether the frequency and osteoblastic differentiation potential of mesenchymal cells from transgenic mice, where a fragment of the α*Sma* promoter directs the expression of green fluorescent protein (GFP), is sexually dimorphic [15], [16]. To determine the influence of sex on osteoclastogenesis, we investigated the differentiation and activity of osteoclast precursors in bone marrow cell cultures from male or female C57BL/6 mice and measured the *Rankl* to *Opg* ratio in calvarial osteoblasts from male or female C57BL/6 mice.

## Materials and Methods

### Bone Histomorphometry

Femurs from 1, 3 and 6 month old male or virgin female C57BL/6 littermate mice were dissected from surrounding tissue, fixed in 70% ethanol, dehydrated and embedded in methyl methacrylate. Static histomorphometry was carried out on 5 µm thick longitudinal sections stained with toluidine blue (Sigma-Aldrich, St. Louis, MO), as described [17]. Bone volume over tissue volume (BV/TV), trabecular number (TbN) and thickness (TbTh), number of osteoblasts per bone perimeter (NOb/BPm), osteoblast and osteoid surface over bone surface (ObS/BS and OS/BS, respectively), number of osteoclasts per bone perimeter (NOc/BPm) and osteoclast and eroded surface over bone surface (OcS/BS and ES/BS, respectively) were measured with an OsteoMeasure morphometry system (Osteometrics, Atlanta, GA) in a defined area between 360 µm and 2160 µm from the growth plate. Dynamic histomorphometry was carried out on sections from mice injected with calcein 20 mg/kg and demeclocycline 50 mg/kg, at an interval of 2 days for 1 month old or 7 days for 3 and 6 month old mice, and sacrificed by CO_2_ inhalation 2 days after demeclocycline administration. Mineralizing surface per bone surface (MS/BS) and mineral apposition rate (MAR) were measured under ultraviolet light, using a triple diamino-2-phenylindole fluorescein set long-pass filter, and used to calculate bone formation rate (BFR). Terminology and units recommended in the 2012 update of the American Society for Bone and Mineral Research Histomorphometry Nomenclature Committee, were used [18].

### αSma-GFP Transgenic Mice

Transgenic C57BL/6 mice where the murine *αSma* promoter directs expression of GFP (*αSma-GFP*) were provided by S. Sato (University of Oklahoma Health Science Center, Oklahoma City, OK) and J. Tsai (National Eye Institute, NIH, Bethesda, MD) [15], [16]. In these mice, a 1.1 kb fragment of the murine *αSma* promoter and genomic regions comprised between the transcription start site and the first 15 base pairs (bp) of exon 2, were cloned upstream of the GFP coding sequence and a 240 bp fragment of the SV40 early polyadenylation sequence.

### Primary Bone Marrow Stromal Cell Cultures and Fluorescent Activated Cell Sorting (FACS)

Two to 4, 1 month old C57BL/6, FVB, C3H/HeJ, BALB/c or *αSma-GFP* transgenic male and female littermate mice were sacrificed by CO_2_ inhalation, and femurs and tibiae dissected aseptically. The proximal epiphysis of the femur or the distal epiphysis of the tibia were removed and bone marrow cells recovered by centrifugation or by flushing with α-minimum essential medium (α-MEM, Life Technologies, Grand Island, NY). Following removal of tissue debris and cellular aggregates by filtration, cell number was determined on a Nikon TMS inverted phase-contrast microscope (Nikon Inc., Melville, NY). Whole bone marrow cell extracts from male and female mice exhibited similar number of cells (Table 1). Cells were seeded at a density of 1.25×10^6^ cells/cm^2^ in α-MEM containing 15% fetal bovine serum (FBS; Atlanta Biologicals, Norcross, GA), which was inactivated by heating to 55°C for 30 min, and grown in a humidified 5% CO_2_ incubator at 37°C. Cells in suspension were removed by replacing culture medium 48 h after seeding and adherent cells were considered bone marrow stromal cells [8]. Four days after seeding, bone marrow stromal cells from C57BL/6, C3H/HeJ and BALB/c mice were exposed to α-MEM supplemented with heat-inactivated 10% FBS, 100 µg/ml ascorbic acid and 5 mM β-glycerophosphate (all from Sigma-Aldrich), to induce osteoblastic differentiation. Bone marrow stromal cells from FVB mice were exposed to culture conditions favoring osteoblastogenesis upon reaching confluence 12 days after seeding. To calculate the frequency of colony forming units positive for alkaline phosphatase (CFU-ALP) and to estimate cell proliferation, cells were seeded at a density of 6×10^5^ cells/cm^2^ and exposed to conditions favoring osteoblastogenesis 7 days after seeding.

**Table 1:** Number of bone marrow cells harvested for from femurs and tibiae of 1 month old male and female C57BL/6, FVB, C3H/HeJ and BALB/c mice.

GeneticBackground	Sex	Bone marrow cellnumber/mouse	p Value
C57BL/6	Males	1.97×10^8^±2.44×10^7^	0.46
	Females	1.99×10^8^±2.24×10^7^	
FVB	Males	1.92×10^8^±1.76×10^7^	0.94
	Females	1.75×10^8^±1.33×10^7^	
C3H/HeJ	Males	1.05×10^8^±1.54×10^7^	0.65
	Females	1.13×10^8^±6.84×10^6^	
BALB/c	Males	1.83×10^8^±2.00×10^7^	0.88
	Females	1.79×10^8^±1.47×10^7^	

Values are average ± SEM, n = 5–10 mice for each sex.

The frequency of bone marrow stromal cells expressing the *αSma-GFP* transgene was determined in cells from male or female *αSma-GFP* transgenic mice, cultured for 7 days, by FACS analysis performed on a LSRII sorter (Benton Dickinson Biosciences, Franklin Lakes, NJ). To enrich for mesenchymal progenitor cells, bone marrow stromal cells expressing the *αSma-GFP* transgene were sorted using BD-FACS Aria II sorter (Benton Dickinson Biosciences), as described [11]. Sorted cells were seeded at a density of 25,000 cells/cm^2^ in α-MEM containing 10% FBS and cultured in a humidified 5% CO_2_ incubator at 37°C. Cells were allowed to expand for 3 days before exposure to conditions that promote osteoblastic differentiation for an additional period of 8 days. Cells that did not express GFP were considered controls.

### Primary Osteoblast-enriched Cell Cultures

The parietal bones of 3 to 5 day old C57BL/6, FVB, C3H/HeJ or BALB/c male or female littermate mice were dissected from the sagittal, lamboid and coronal sutures to avoid contamination from fibroblastic and chondrogenic cells. Dissected bones were exposed to type-II collagenase from *Clostridium histolyticum* (Worthington Biochemical Corp., Lakewood, NJ) pretreated with N-α-tosyl-L-lysyl-chloromethylketone 17 µg/ml (Calbiochem, La Jolla, CA), to protect cells from damage due to clostripain activity [19]. Bones were digested for 20 min at 37°C, cells extracted in five consecutive reactions and cells from the last 3 digestions were pooled and seeded at a density of 60,000 cells/cm^2^, as described [20]. Sex was determined prior to sacrifice by polymerase chain reaction (PCR) analysis of genomic DNA with specific primers (Integrated DNA Technologies; IDT, Coralville, IA) for sex-determining region Y and fatty acid-binding protein 1 (Table 2). Osteoblast-enriched cells were cultured in Dulbecco’s modified Eagle’s medium (DMEM) supplemented with non-essential amino acids (both from Life Technologies), 20 mM HEPES (Sigma-Aldrich), 100 µg/ml ascorbic acid and 10% heat-inactivated FBS, in a humidified 5% CO_2_ incubator at 37°C. To promote maturation, confluent osteoblasts were exposed to DMEM supplemented with 10% heat-inactivated FBS, 100 µg/ml ascorbic acid and 5 mM β-glycerophosphate.

**Table 2:** Primers used for PCR.

Gene	Strand	Primer Sequence	Product length (base pairs)
*Sry*	Fwd	5′-GAGAGCATGGAGGGCAT-3′	400
	Rev	5′-GAGTACAGGTGTGCAGCTC-3′	
*Fabp1*	Fwd	5′-TGGACAGGACTGGACCTCTGCTTTCC-3′	200
	Rev	5′-TAGAGCTTTGCCACATCACAGGTCAT-3′	

Forward (Fwd) and reverse (Rev) primers used for assessing sex of newborn mice.

### Primary Osteoclast-like Cell Cultures and Pit Formation Assay

Bone marrow cells were harvested from 4 male and 4 female littermate C57BL/6 mice at one month of age and seeded in α-MEM containing 15% heat-inactivated FBS at a density of 1×10^6^ cells/cm^2^, and maintained in a humidified 5% CO_2_ incubator at 37°C. To induce expression of Rankl by adherent osteoblastic cells and differentiation of mononuclear precursors into osteoclast-like cells, cultures were exposed for 7 days to 1,25 dihydroxyvitamin D_3_, dissolved in 100% Et-OH and diluted to a concentration of 10 nM in α-MEM containing 15% heat-inactivated FBS [21], [22]. Parallel control cultures were exposed to an equal volume of 100% Et-OH. To determine bone resorptive activity, cells were seeded on Osteo Assay Surface (Corning Life Sciences, Tewksbury, MA), cultured for 7 days under conditions favoring osteoclastogenesis and removed with bleach before von Kossa staining of the culture surface. Images of the stained surfaces were acquired with a Coolpix 995 digital camera (Nikon Inc., Melville, NY), imported in ImageJ v 1.48d (National Institutes of Health, Bethesda, MD) [23] and converted to 8-bit grayscale. Identical thresholds of gray intensity were set below the background of each image analyzed and resorbed area was defined as the area occupied by pixels with values of gray intensity below the preset threshold, determined with the measure function of ImageJ.

### Animal Care and Use

Mice were euthanized by CO_2_ inhalation. Experimental protocols were approved by the Institutional Animal Care and Use Committees of Saint Francis Hospital and Medical Center (Protocol Number 10), and of the University of Connecticut Health Center (100490-0815).

### Cytochemical Assays

Alkaline phosphatase activity was measured in 0.5% Triton X-100 cell extracts (Sigma-Aldrich) by the hydrolysis of *p-*nitrophenyl phosphate to *p*-nitrophenol (Sigma-Aldrich) and total protein content was determined by DC protein assay (Bio-Rad, Hercules, CA) according to manufacturer’s instructions. To detect mineralized nodules, bone marrow stromal cells fixed with 3.7% formaldehyde in phosphate buffered saline were stained with 2% alizarin red (all from Sigma-Aldrich) [24]. To estimate the frequency of osteoblastic progenitors, bone marrow stromal cells were fixed and stained with the leukocyte alkaline phosphatase kit (Sigma-Aldrich), in accordance with manufacturer’s instructions [25]. The frequency of CFU-ALP was calculated by normalizing the number of clusters of 20 or more cells stained for alkaline phosphatase for the number of cells seeded for cm^2^ [26]. Biochemically active bone marrow stromal cells were estimated by measuring the product of 3-[4,5-Dimethylthiazol-2-yl]-2,5-diphenyltetrazolium bromide (MTT) reduction to formazan with the MTT cell proliferation assay kit (R&D Systems, Minneapolis, MN), according to manufacturer’s instructions [27]. To determine the number of osteoclast-like cells, bone marrow cells cultures were stained with the leukocyte acid phosphatase kit (Sigma-Aldrich), according to manufacturer’s instruction, and stained cells containing more than three nuclei were counted [28].

### Quantitative Reverse Transcription-PCR (qRT-PCR)

Total RNA was extracted with the RNeasy mini kit, according to manufacturer’s instructions (Qiagen, Valencia, CA). Subsequently, 0.5 µg of total RNA were reverse-transcribed using the iScript cDNA synthesis kit (BioRad), according to manufacturer’s instructions, and amplified in the presence of specific primers (IDT; Table 3) and iQ SYBR Green Supermix (BioRad) at 55°C for 35 cycles [29], [30]. PCR efficiency and RNA copy number were estimated by comparison to a standard curve generated by parallel amplification of a dilution series of cDNA for alkaline phosphatase liver/bone/kidney (*Alpl*) and ribosomal protein l38 (*Rpl38*; both from American Type Culture Collection, Manassas, VA), calcitonin receptor (*Calcr*), cathepsin K (*Ctsk*), osteoclast associated receptor (*Oscar*), *Opg* and tartrate resistant acid phosphatase (*Trap*; all from Thermo Scientific, Pittsburgh, PA), *Osteocalcin* and runt-related transcription factor 2 (*Runx2*; both from J.B. Lian, University of Vermont, Burlington, VT) and *Rankl* (Source BioScience, Nottingham, UK) [31]. Fluorescence was monitored at the annealing step of every reaction cycle and specificity of the reaction confirmed by presence of a single peak in the melt curve analysis of PCR products. mRNA levels corrected for *Rpl38* mRNA expression are reported as ratios relative to corrected mRNA levels in cells from male mice at the initiation of the culture [32].

**Table 3:** Primers used for qRT-PCR.

Gene	Strand	Primer Sequence	GenBank Accession Numbers
*Alpl*	Fwd	5′-TGGTATGGGCGTCTCCACAGTAACC-3′	NM_007431
	Rev	5′-CTTGGAGAGGGCCACAAAGG-3′	
*Calcr*	Fwd	5′-TCTGAGAAACTGCAAAATGCGTAC-3′	NM_001042725; NM_007588.2
	Rev	5′-AGCAACCAAAGCAGCAATCG-3′	
*Ctsk*	Fwd	5′-AGATATTGGTGGCTTTGGAA-3′	NM_007802
	Rev	5′-AACGAGAGGAGAAATGAAACA-3′	
*Opg*	Fwd	5′-CAGAAAGGAAATGCAACACATGACAAC-3′	NM_011613
	Rev	5′-GCCTCTTCACACAGGGTGACATC-3′	
*Oscar*	Fwd	5′-TTGCTATTACCACACGCCTTCT-3′	NM_175632
	Rev	5′-CAAGGAGCCAGAACCTTCGA-3′	
*Osteocalcin*	Fwd	5′-GACTCCGGCGCTACCTTGGGTAAG-3′	NM_001037939
	Rev	5′-CCCAGCACAACTCCTCCCTA-3′	
*Rankl*	Fwd	5′-TATAGAATCCTGAGACTCCATGAAAAC-3′	NM_009399
	Rev	5′-CCCTGAAAGGCTTGTTTCATCC-3′	
*Runx2*	Fwd	5′-CGCACCGACAGTCCCAACTTCCTG-3′	NM_001145920; NM_001146038;
	Rev	5′-CACGGGCAGGGTCTTGTTG-3′	NM_001271631; NM_009820
*Trap*	Fwd	5′-GACAAGAGGTTCCAGGAGAC-3′	NM_001102404; NM_001102405;
	Rev	5′-TTCCAGCCAGCACATACC-3′	NM_007388
*Rpl38*	Fwd	5′-AGAACAAGGATAATGTGAAGTTCAAGGTTC-3′	NM_001048057; NM_001048058;
	Rev	5′-CTGCTTCAGCTTCTCTGCCTTT-3′	NM_023372

Forward (Fwd) and reverse (Rev) primers used for measuring changes in gene expression by qRT-PCR. GenBank accession numbers indicate transcript variants with homologous sequences to primers.

### Statistical Analysis

Data are expressed as means ± SEM. To determine whether the effect of sex and time on parameters of bone histomorphometry was statistically significant, we accounted for the intracorrelation between mice from the same experiment. To this end, generalized linear models were estimated with a generalized estimating equation; exchangeable correlation structures were used. Independent variables in the models were sex and time, and all models included the interaction between sex and time. Dependent variables were static and dynamic parameters of bone histomorphometry [33]. Calculations were performed with SAS v 9.3 (SAS Institute, Cary, NC). Statistical differences in results from *in vitro* studies were determined by Student’s *t* test or analysis of variance with Schaeffe’s post-hoc analysis for pairwise or multiple comparisons, respectively, by using PASW software v 18.0.0 (IBM, Armonk, NY) [34].

## Results

### Histomorphometry of Distal Femur from C57BL/6 Mice

**Figure 1:**
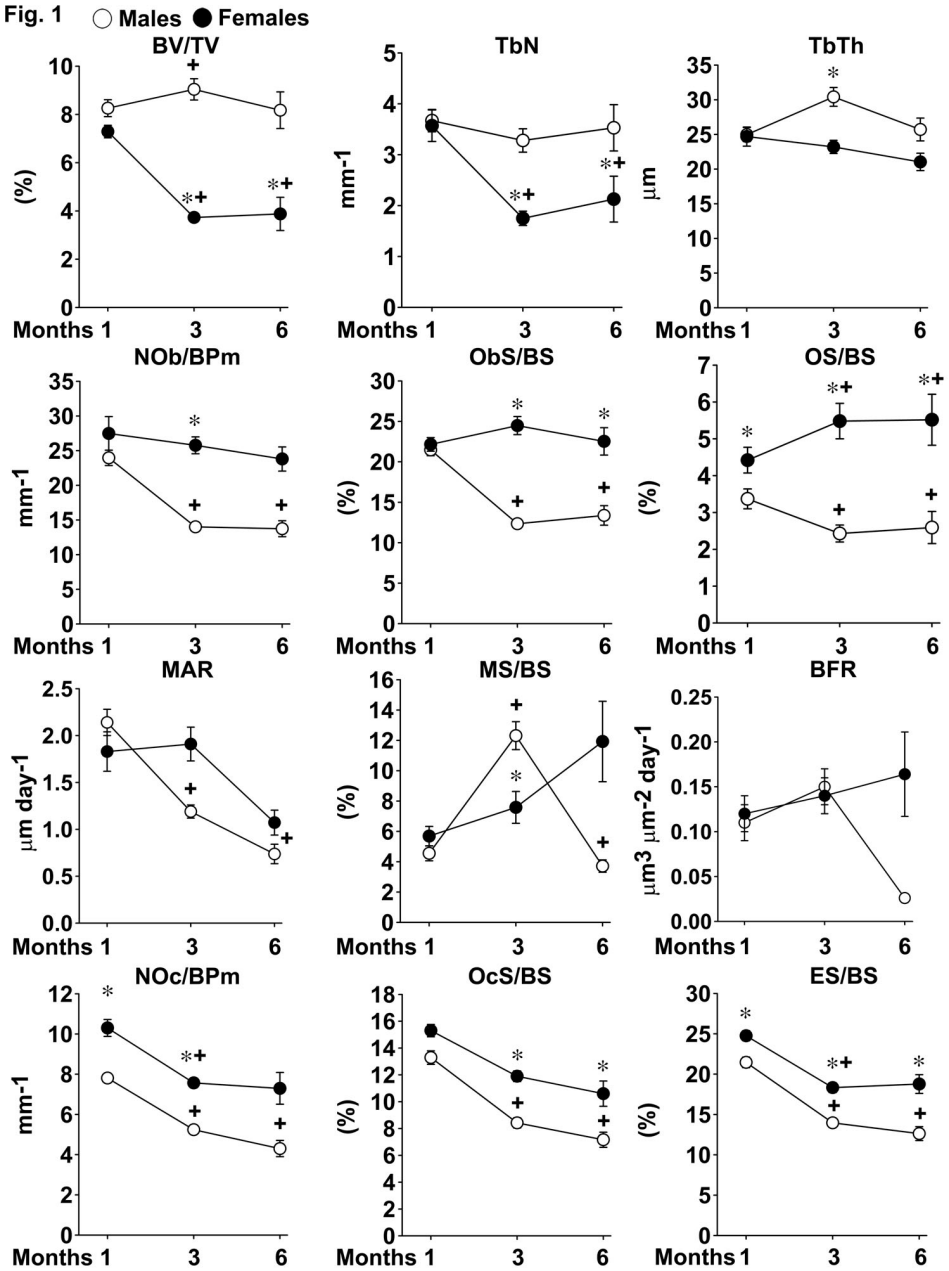
Femoral bone histomorphometry of C57BL/6 mice. Static and dynamic parameters of bone formation and resorption were determined by histomorphometric analysis of trabecular bone from femurs of 1, 3 and 6 old month old male or female littermate C57BL/6 mice. Values are means ± SEM from 14 to 66 mice. *Significantly different between males and females, *p*<0.025. +Significantly different from 1 month mice of the same sex, *p*<0.025.

To establish whether the skeleton of maturing mice exhibits changes related to sex, we measured static and dynamic parameters of bone histomorphometry from 1, 3 and 6 month old male and female littermate C57BL/6 mice. Selected control histomorphometric data were extracted from previously published studies aimed at defining the phenotype of mice harboring the global or conditional inactivation of various genes (Canalis and Zanotti, unpublished observations) [35]–[39]. Trabecular bone volume, number and thickness were not different between male and female mice at 1 month of age. Trabecular bone volume remained stable in male mice until 6 months of age, but declined in female littermates at 3 months of age, and then it stabilized so that 3 and 6 month old female mice exhibited lower bone volume than male littermates. Trabecular number and thickness followed the same trend, although the differences in trabecular thickness between male and female mice at 6 months of age did not achieve statistical significance (Fig. 1). Osteoblast number and surface were not sexually dimorphic at 1 month of age, but declined as male, but not female, mice aged, so that female mice had higher osteoblast number and surface than male littermates at 3 and 6 months of age. Osteoid surface was higher in female mice than in male littermates, remained stable in maturing female mice and declined in maturing male littermates (Fig. 1). Parameters of bone formation were not significantly different between sexes as mice matured, with the exception of mineralizing surface per bone surface, which was higher in 3 month old male mice than in female littermates (Fig. 1). Male mice exhibited fewer osteoclasts and less eroded surface than female littermates at 1 month of age. Indices of bone resorption declined to a similar extent in maturing mice of both sexes, so that they were higher in 3 and 6 month old female mice than in male littermates, although the difference in osteoclast number was not statistically significant at 6 months of age (Fig. 1). Histomorphometric analysis of femurs from 1 and 3 month old male and female littermate FVB mice revealed similar trends, although differences between sexes did not achieve statistical significance (data not shown). These findings indicate that trabecular bone volume declines more rapidly in female than in male C57BL/6 mice possibly due to higher bone resorption in female mice than in male littermates [2].

### Differentiation of Bone Marrow Stromal Cells from C57BL/6 Mice

**Figure 2:**
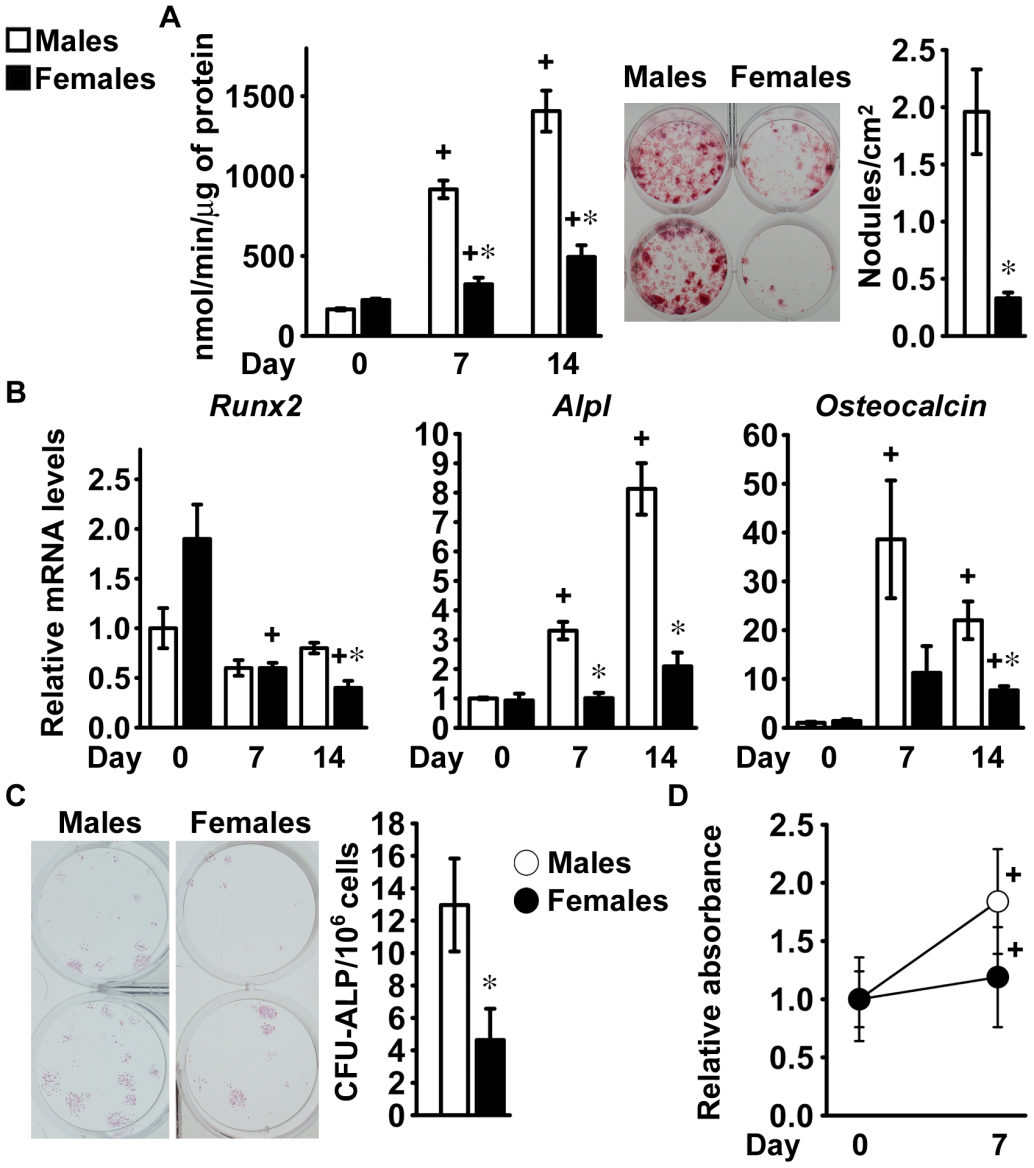
Differentiation of bone marrow stromal cells from C57BL/6 mice. Bone marrow stromal cells were harvested from 1 month old male (white bars) or female (black bars) littermate C57BL/6 mice. Cells were cultured for 4 (panels A and B) or 7 days (panels C and D) before exposure to conditions favoring osteoblastic differentiation (Day 0). In panel A, cells were extracted with Triton X-100 at the indicated times for determination of alkaline phosphatase activity, expressed as nanomoles of p-nitrophenol/min/µg of total protein. Values are means ± SEM and represent 4 technical replicates. Parallel cultures were fixed at 14 days and stained with alizarin red to detect mineralized nodules, which were counted and expressed as number of nodules for cm^2^. Values are means ± SEM and represent 6 technical replicates. Cells were obtained from 4 male and 3 female mice. Two representative cultures are shown. In panel B, total RNA was extracted at the indicated times and amplified by qRT-PCR. Data are expressed as ratio of *Runx2*, *Alpl* and *Osteocalcin* copy number, corrected for *Rpl38* expression, relative to corrected expression in cells from male mice at Day 0. Values are means ± SEM and represent 4 technical replicates of cultures from 4 mice for each sex. In panel C, cells cultured for 14 days were fixed and stained with alkaline phosphatase to detect cluster of cells committed to the osteoblastic lineage, which were counted and expressed as number of CFU-ALP for 10^6^ cells. Values represent means ± SEM of independent cultures from 4 mice for each sex. In panel D, cells were exposed to MTT at the indicated times for determination of cell viability, and data are expressed as ratio of absorbance at 595 nm corrected by subtraction of absorbance at 650 nm, relative to corrected absorbance in cells at Day 0. Values are means ± SEM from 3 independent experiments. +Significantly different from cells of the same sex at Day 0, *p*<0.05.

The higher number of osteoblasts in mature female mice did not translate in higher bone formation relative to mature male mice and was insufficient to compensate for the enhanced bone resorption so that cancellous bone declined in female mice. Therefore, we explored whether there were differences in the potential for osteoblastogenesis between male and female C57BL/6 mice. A progressive increase in alkaline phosphatase activity and in the formation of mineralized nodules was observed in bone marrow stromal cells from both sexes, demonstrating that osteoblastic differentiation had occurred (Fig. 2A–B). At 7 and 14 days of culture, alkaline phosphatase activity was 70% lower in cells from female mice than in those from male littermates (Fig. 2A). Accordingly, cultures from female mice formed fewer mineralized nodules than cultures from male mice, suggesting reduced osteoblastic differentiation (Fig. 2A). As osteoblastic differentiation progressed, *Runx2* expression remained stable in cells from male mice and declined modestly in cells from female littermates. Conversely, *Alpl* and *Osteocalcin* mRNA levels increased to a greater extent in cultures from male than female mice, confirming that cells from female C57BL/6 mice display less osteoblastic differentiation potential than cells from male littermates (Fig. 2B).

To investigate possible mechanisms responsible for the sexually dimorphic osteoblastic differentiation potential of bone marrow stromal cells, frequency of osteoblast progenitors and cell proliferation were measured in cells seeded at a reduced density, to allow for the formation of discrete cell clusters. Bone marrow stromal cells from female C57BL/6 mice displayed reduced CFU-ALP frequency when compared to cells from male littermates and cell number increased in cultures from male mice to a greater extent than in those from female mice (Fig. 2C–D). These findings indicate that differences related to the number of osteoblast progenitors and cell proliferation contribute to the sexually dimorphic osteoblastic differentiation potential of bone marrow stromal cells from C57BL/6 mice.

### Differentiation of Bone Marrow Stromal Cells from FVB Mice

**Figure 3:**
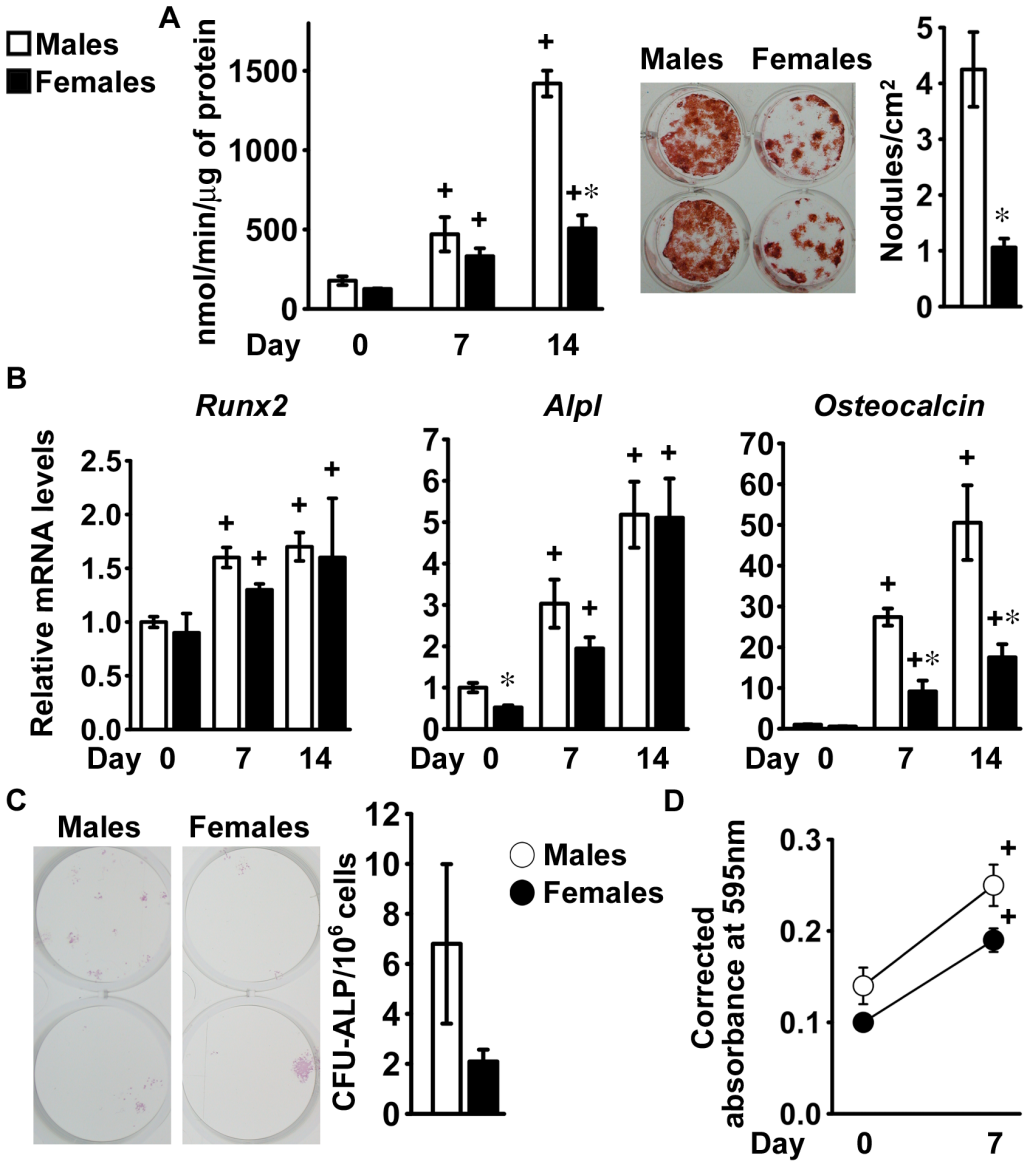
Differentiation of bone marrow stromal cells from FVB mice. Bone marrow stromal cells were harvested from 1 month old male (white bars) or female (black bars) littermate FVB mice. At confluence (Day 0; panels A and B) or 7 days after seeding (Day 0; panels C and D), cells were exposed to conditions favoring osteoblastic differentiation. In panel A, cells were extracted with Triton X-100 at the indicated times for determination of alkaline phosphatase activity, expressed as nanomoles of p-nitrophenol/min/µg of total protein. Values are means ± SEM and represent 4 technical replicates. Parallel cultures were fixed at 14 days and stained with alizarin red to detect mineralized nodules, which were counted and expressed as number of nodules for cm^2^. Values are means ± SEM and represent 6 technical replicates. Cells were obtained from 3 mice for each sex. Two representative cultures are shown. In panel B, total RNA was extracted at the indicated times, and amplified by qRT-PCR. Data are expressed as ratio of *Runx2*, *Alpl* and *Osteocalcin* copy number, corrected for *Rpl38* expression, relative to corrected expression in cells from male mice at Day 0. Values are means ± SEM and represent 3 to 4 technical replicates of cultures from 4 mice for each sex. In panel C, cells cultured for 14 days were fixed and stained with alkaline phosphatase to detect cluster of cells committed to the osteoblastic lineage, which were counted and expressed as number of CFU-ALP for 10^6^ cells. Values represent means ± SEM of independent cultures from 3 male and 4 female mice. In panel D, cells were exposed to MTT at the indicated times for determination of cell viability, expressed as absorbance at 595 nm corrected by subtraction of absorbance at 650 nm. Values represent means ± SEM of independent cultures from 3 male and 4 female mice. *Significantly different between males and females, *p*<0.05. +Significantly different from cells of the same sex at Day 0, *p*<0.05.

To ensure that the cellular phenotype was not selective to the C57BL/6 genetic background, osteoblastogenesis was studied in cells from male and female littermate FVB mice. Alkaline phosphatase activity increased following 7 and 14 days of culture in conditions favoring osteoblastogenesis and formation of mineralized nodules was observed at 14 days, demonstrating osteoblastic differentiation (Fig. 3A). In agreement with the results from C57BL/6 cells, following 14 days of culture alkaline phosphatase activity and mineralized nodule formation were greater in cells from male than from female mice (Fig. 3A).

Culture conditions favoring osteoblastogenesis resulted in a limited increase in *Runx2* expression. *Alpl* transcripts levels increased to a lesser extent in cells from female mice than in cells from male littermates, up to 7 days after confluence (Fig. 3B), resulting in a difference in alkaline phosphatase activity as osteoblastic cells matured. *Osteocalcin* expression was minimal in confluent cells and increased to a greater extent in cultures from male than those from female mice (Fig. 3B). Consequently, osteocalcin mRNA levels were about 70% lower in cells from female mice after 14 days of culture indicating a lesser degree of osteoblastic differentiation by these cells.

Confirming the results obtained in the C57BL/6 genetic background, cultures from female mice displayed a tendency (*p* = 0.086) to a reduced CFU-ALP frequency in comparison to cells from male littermates, although cells from either sex proliferated to a similar extent (Fig. 3C–D).

### Differentiation of Bone Marrow Stromal Cells from C3H/HeJ and BALB/c Mice

**Figure 4:**
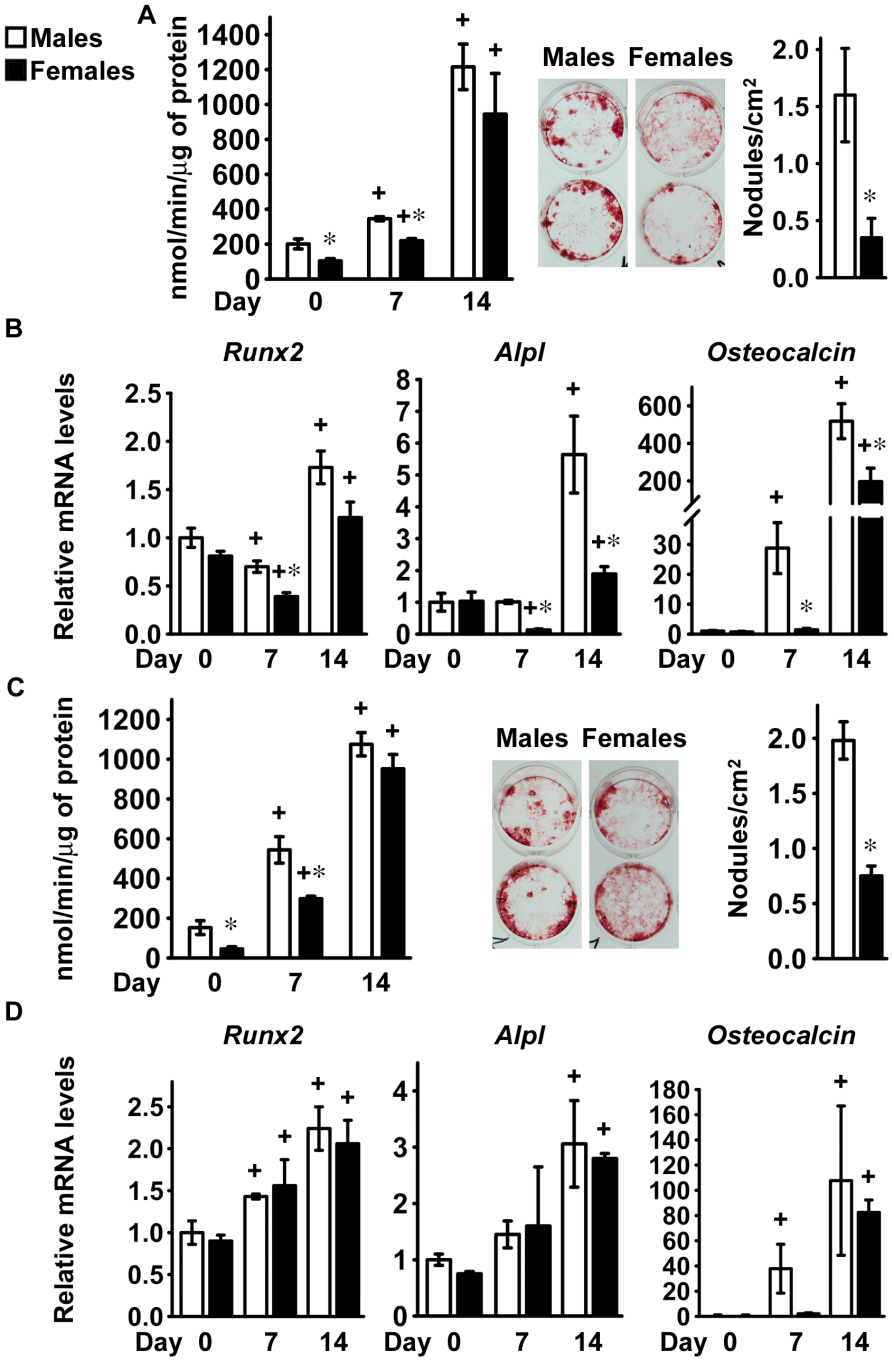
Differentiation of bone marrow stromal cells from C3H/HeJ and BALB/c mice. Bone marrow stromal cells were harvested from 1 month old male (white bars) or female (black bars), littermate C3H/HeJ (panels A and B) or BALB/c (panels C and D) mice. Cells were cultured for 4 days before exposure to conditions favoring osteoblastic differentiation (Day 0). In panels A and C, cells were extracted with Triton X-100 at the indicated times for determination of alkaline phosphatase activity, expressed as nanomoles of p-nitrophenol/min/µg of total protein. Parallel cultures were fixed at 14 days and stained with alizarin red to detect mineralized nodules, which were counted and expressed as number of nodules for cm^2^. Values represent means ± SEM of independent cultures from 4 mice for each sex. In panels B and D, total RNA was extracted at the indicated times and amplified by qRT-PCR. Data are expressed as ratio of *Runx2*, *Alpl* and *Osteocalcin* copy number, corrected for *Rpl38* expression, relative to corrected expression in cells from male mice at Day 0. Values represent means ± SEM of independent cultures from 4 mice for each sex. *Significantly different between males and females, *p*<0.05. +Significantly different from cells of the same sex at Day 0, *p*<0.05.

To assess the impact of genetic factors on the sexually dimorphic potential for osteoblastogenesis of bone marrow stromal cells, we also investigated osteoblastic differentiation in bone marrow stromal cells from C3H/HeJ and BALB/c mice. Cells from mice of both genetic backgrounds exhibited increased alkaline phosphatase activity as the culture progressed and formed mineralized nodules after 14 days of culture, documenting that osteoblast differentiation had occurred (Fig. 4A–C). In the initial phase of the culture, cells from female C3H/HeJ and BALB/c mice exhibited lower alkaline phosphatase activity than cells from male mice, although after 14 days of culture no differences between sexes were noticed. In agreement with the results obtained in cells from FVB and C57BL/6 mice, fewer mineralized nodules were observed in cultures from female than from male C3H/HeJ and BALB/c mice (Fig. 4A–C).

As the osteoblastic phenotype developed in bone marrow stromal cells from C3H/HeJ mice, cells from female mice displayed a modest and transient reduction in *Runx2* mRNA levels. In accordance with findings in C57BL/6 mice, *Alpl* and *Osteocalcin* transcript levels increased in cultures from male mice to a greater extent than in cultures from female littermates (Fig. 4B). In bone marrow stromal cells from BALB/c mice, osteoblastic differentiation was confirmed by increased expression of *Alpl* and *Osteocalcin*, although no sexual dimorphism was detected (Fig. 4D). These results demonstrate that genetic factors determine the magnitude of the effect of sex on the osteoblastic differentiation potential of bone marrow stromal cells.

### Differentiation of Bone Marrow Stromal Cells Expressing the αSma-GFP Transgene

**Figure 5:**
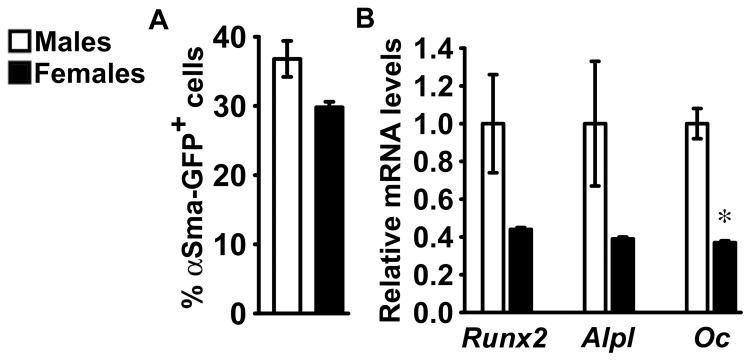
Sorting and differentiation of bone marrow stromal cells from αSma-GFP transgenic mice. Bone marrow stromal cells were harvested from 1 month old male (white bars) or female (black bars) littermate *αSma-GFP* transgenic mice and adherent cells that retained the individual identity of the donor mouse were cultured for 7 days. In panel A, the ratio of cells expressing αSma-GFP (αSma-GFP^+^) over total cells was determined by FACS analysis. Values represent means ± SEM of independent cultures from 4 mice for each sex. In panel B, αSma-GFP^+^ were sorted by FACS and equal amounts of cells for individual mouse seeded and cultured for 8 days under conditions favoring osteoblastic differentiation. Total RNA was extracted and amplified by qRT-PCR and data are expressed as ratio of *Runx2*, *Alpl* and *Osteocalcin* (*Oc*) copy number, corrected for *Rpl38* expression, relative to corrected expression in cells from male mice. Values represent means ± SEM of independent cultures from 2 mice for each sex. *Significantly different between males and females, *p*<0.05.

To determine whether the frequency of mesenchymal cells in bone marrow stromal cell cultures is sexually dimorphic and to study osteoblastogenesis in cultures enriched in mesenchymal cells, bone marrow cells were harvested from male or female littermate *αSma-GFP* transgenic C57BL/6 mice. In agreement with findings in bone marrow stromal cells from C57BL/6 and FVB mice, FACS analysis revealed a greater frequency of cells expressing GFP in cultures from male than in those from female *αSma-GFP* transgenic mice, although the difference did not reach statistical significance (Fig. 5A). Following sorting, cells were seeded so that cultures from male and female mice contained equal numbers of cells expressing the *αSma-GFP* transgene. Cells not expressing GFP failed to adhere to the culture substrate, whereas cells expressing GFP adhered to the substrate and grew to confluence. After 8 days under conditions favoring osteoblastogenesis, cells from female mice expressed *Runx2*, *Alpl*, *Osteocalcin* to a lesser degree than cells from male littermates, although only the effect on *Osteocalcin* mRNA levels reached statistical significance (Fig. 5B).

### Differentiation of Calvarial Osteoblasts

**Figure 6:**
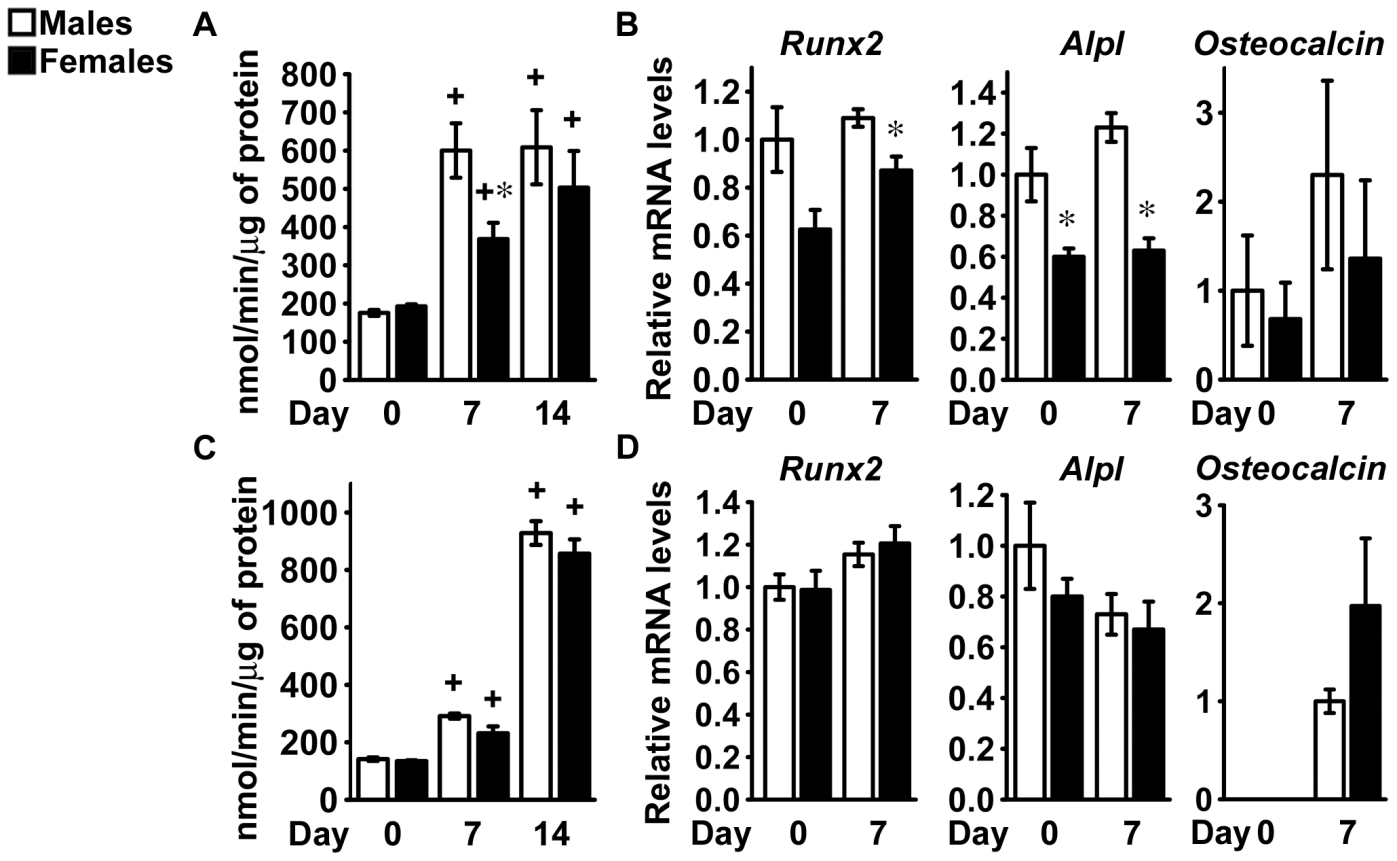
Differentiation of calvarial cells from C57BL/6 and FVB mice. Primary calvarial osteoblasts were harvested from three to five day old male (white bars) or female (black bars) littermate C57BL/6 (panels A and B) or FVB (panels C and D) mice. Confluent cells were cultured for 14 days under conditions favoring osteoblastic differentiation. In panels A and C, cells were extracted at the indicated times with Triton X-100 for determination of alkaline phosphatase activity, expressed as nanomoles of p-nitrophenol/min/µg of total protein. Values are means±SEM, n = 6. In panels B and D, total RNA was extracted at the indicated times and amplified by qRT-PCR. Data are expressed as ratio of *Runx2*, *Alpl* and *Osteocalcin* copy number, corrected for *Rpl38* expression, relative to corrected expression in cells from male mice at Day 0. Values are means ± SEM, n = 4. *Significantly different between males and females, *p*<0.05. +Significantly different from cells of the same sex at Day 0, *p*<0.05.

**Figure 7:**
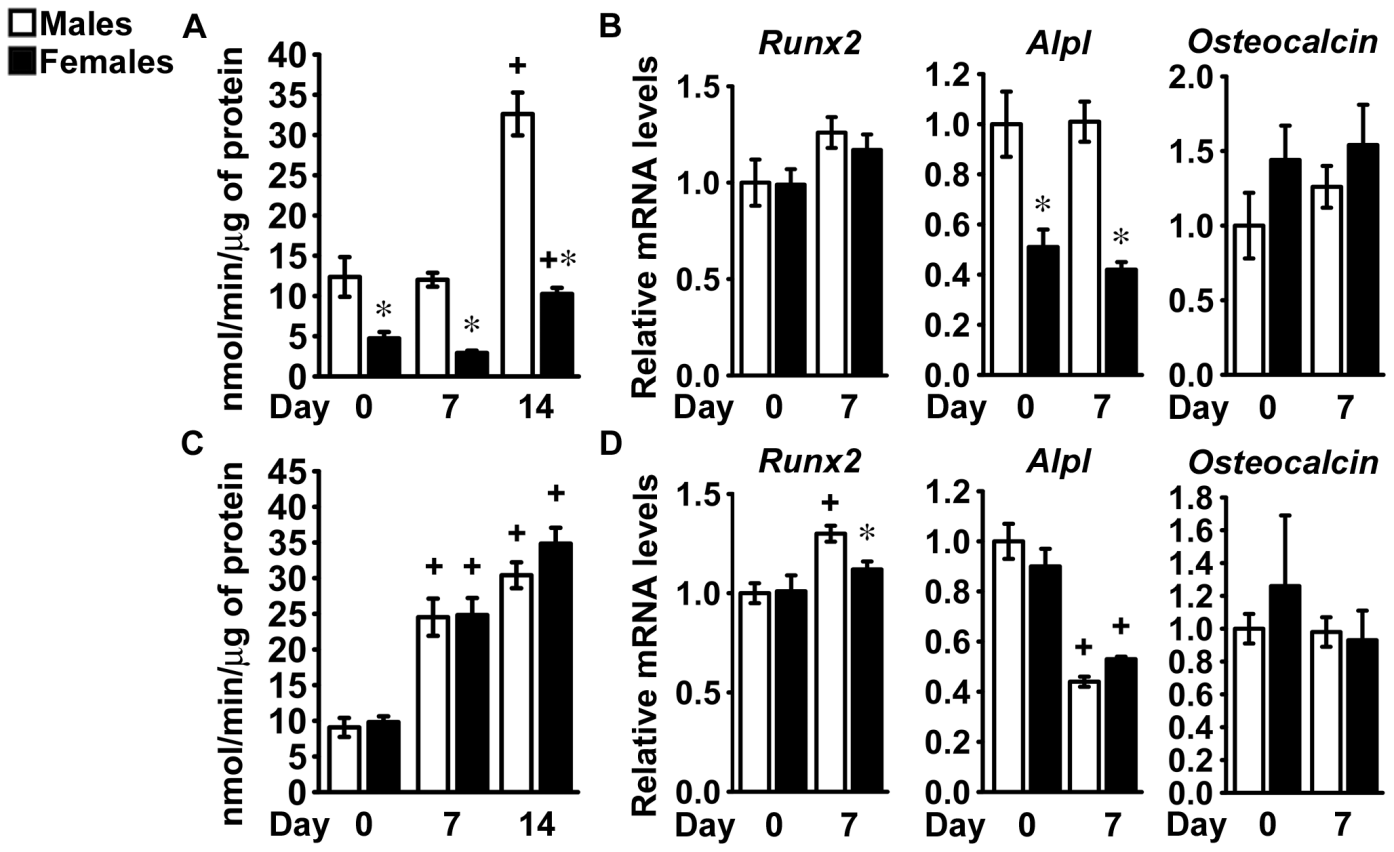
Differentiation of calvarial cells from C3H/HeJ and BALB/c mice. Primary calvarial osteoblasts were harvested from three to five day old male (white bars) or female (black bars) littermate C3H/HeJ (panels A and B) or BALB/c (panels C and D) mice. Confluent cells were cultured for 14 days under conditions favoring osteoblastic differentiation. In panels A and C, cells were extracted at the indicated times with Triton X-100 for determination of alkaline phosphatase activity, expressed as nanomoles of p-nitrophenol/min/µg of total protein. Values are means ± SEM, n = 6. In panels B and D, total RNA was extracted at the indicated times and amplified by qRT-PCR. Data are expressed as ratio of *Runx2*, *Alpl* and *Osteocalcin* copy number, corrected for *Rpl38* expression, relative to corrected expression in cells from male mice at Day 0. Values are means ± SEM, n = 4. *Significantly different between males and females, *p*<0.05. +Significantly different from cells of the same sex at Day 0, *p*<0.05.

To investigate whether sexual dimorphism affects the function of mature osteoblastic cells, we cultured primary calvarial osteoblasts harvested from C57BL/6, FVB, C3H/HeJ and BALB/c mice. In cells from C57BL/6 mice, alkaline phosphatase activity increased as the culture progressed and the magnitude of the effect was larger in osteoblasts from male than female mice, so that cells from female mice exhibited lower alkaline phosphatase activity than cells from male littermates (Fig. 6A). Accordingly, cells from female mice expressed lower levels of *Alpl* transcripts than cells from male mice (Fig. 6B). Although cells from female mice exhibited a tendency toward reduced expression of *Runx2* in comparison to cells from male littermates, *Osteocalcin* expression was not different between cells from mice of both sexes (Fig. 6B). Cultures from male and female FVB mice progressed to a similar extent toward a mature osteoblastic phenotype, indicating that osteoblast function is not sexually dimorphic in osteoblasts from FVB mice (Fig. 6C and D). In agreement with the findings in C57BL/6 osteoblasts, alkaline phosphatase activity increased to a greater extent in cells from C3H/HeJ male mice than in those from female littermates, and osteoblasts from female mice exhibited lower *Alpl* expression than cells from male mice (Fig. 7A and B). As observed in cells from FVB mice, sex did not determine osteoblast function in cells from BALB/c mice (Fig. 7C and D). These results indicate that sex determines *Alpl* expression and activity in osteoblasts from C57BL/6 and C3H/HeJ mice, whereas it has no impact on the function of mature osteoblasts from FVB and BALB/c mice.

### Osteoclastogenesis in Cell Cultures from C57BL/6 Mice

**Figure 8:**
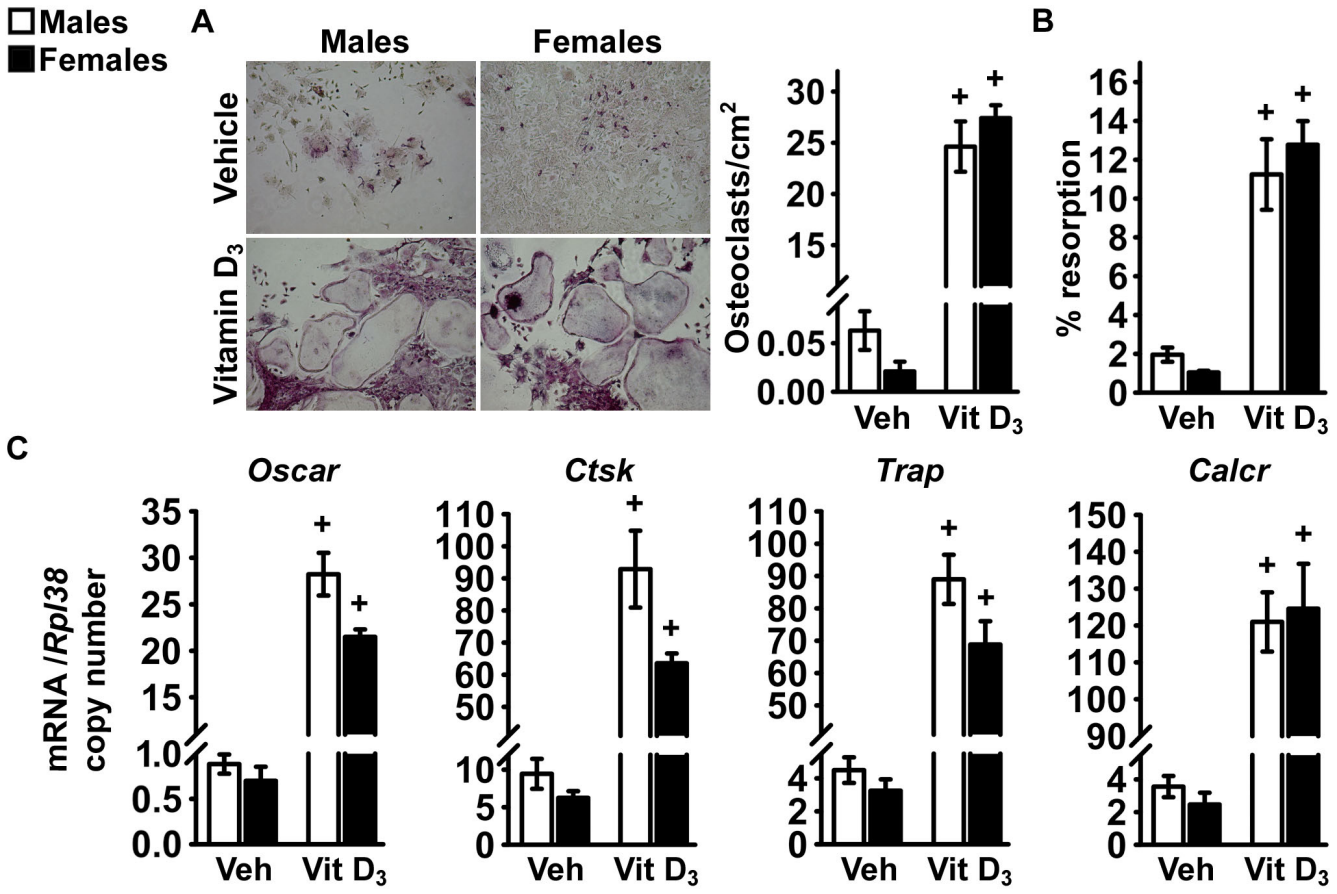
Osteoclastogenesis in bone marrow cell cultures from C57BL/6 mice. Bone marrow cells were harvested from 1 month old male (white bars) or female (black bars) littermate C57BL/6 mice and exposed for 7 days to 10 nM 1,25 dihydroxyvitamin D_3_ (Vit D_3_) or control vehicle (Veh). In panel A, cultures were stained for Trap and stained cells containing more than three nuclei were considered osteoclast-like cells. Data are expressed as number of osteoclasts for cm^2^. Values are means ± SEM, n = 4. In panel B, cells plated on Osteo Assay Surface were removed with bleach, the culture substrate stained by Von Kossa, and digital pictures acquired to determine the extent of resorbed area. Data are expressed as % of resorbed area over total culture area. Values are means ± SEM, n = 4. In panel C, total RNA was extracted and amplified by qRT-PCR. Data are expressed as copy number of *Oscar*, *Ctsk*, *Trap* and *Calcr*, corrected for *Rpl38* copy number. Values represent means ± SEM of independent cultures from 4 mice for each sex. +Significantly different from cells of the same sex exposed to vehicle, *p*<0.05.

**Figure 9:**
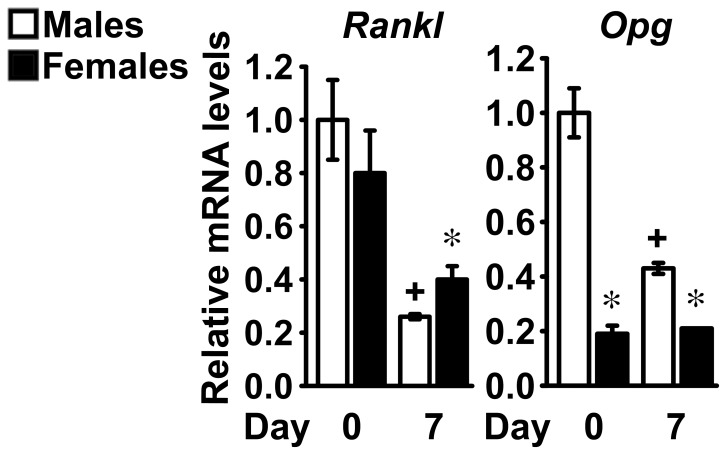
Expression of *Rankl* and *Opg* in calvarial cells from C57BL/6 mice. Primary calvarial osteoblasts were harvested from three to five day old male (white bars) or female (black bars) littermate C57BL/6 mice. Confluent cells were cultured for 7 days under conditions favoring osteoblastic differentiation; total RNA was extracted at the indicated times and amplified by qRT-PCR. Data are expressed as ratio of *Rankl* and *Opg* copy number, corrected for *Rpl38* expression, relative to corrected expression in cells from male mice at Day 0. Values are means ± SEM, n = 4. *Significantly different between males and females, *p*<0.05. +Significantly different from cells of the same sex at Day 0, *p*<0.05.

To investigate the mechanisms that determine higher levels of bone resorption in female mice than in male littermates, we assessed the formation and activity of osteoclast-like cells in primary bone marrow cells from C57BL/6 mice. Under basal conditions, formation of multinucleated osteoclast-like cells was minimal in cultures from both sexes. Exposure of the cultures to 1,25 dihydroxyvitamin D_3_ for 7 days increased the number of multinucleated osteoclast-like cells, induced resorptive activity and increased expression of *Oscar*, *Ctsk*, *Trap* and *Calcr* to a similar extent in cells from male and female mice (Fig. 8). The effect of sex on expression of sex steroids was assessed, but no changes related to sex on the expression of the androgen receptor were observed and transcript levels of estrogen receptor 1 and 2 were not detected (Tables S1 and S2). We tested whether sex determines expression of *Rankl* and *Opg* in osteoblasts from C57BL/6 mice. *Rankl* transcript levels were similar in confluent cultures from mice of both sexes, but were higher in cells from female mice than in those from male littermates after 7 days of culture (Fig. 9). *Opg* expression was 80% lower in cells from female mice than in those from male littermates at confluence and a similar difference, albeit less pronounced, was present after 7 days of culture (Fig. 9). These findings demonstrate that the ratio of *Rankl* to *Opg* is lower in cells from female mice than in those from male littermates, possibly explaining the higher levels of bone resorption observed in female mice *in vivo*.

## Discussion

In the present work, we confirmed that cancellous bone volume declines in maturing female C57BL/6 mice, whereas it remains stable in male littermates. Femoral length was not different between male and female mice (), indicating that sex had no bearing on skeletal growth. Bone resorption that exceeds the capacity of osteoblasts to form new bone seems to explain the decline in cancellous bone observed in female mice. *In vitro* studies in osteoblastic cells from C57BL/6 mice suggest that this effect is secondary to differential expression of *Rankl* and *Opg* by mature osteoblasts and not mediated by the expression of sex steroid receptors.

Parameters of bone formation were not sexually dimorphic, although female mice exhibited a higher number of osteoblasts than male mice, suggesting that compensatory mechanisms acting on osteoblast differentiation or function preclude sex from having an impact on bone formation. Accordingly, *in vitro* studies revealed that bone marrow stromal cell cultures from female mice exhibit reduced osteoblastic differentiation potential compared to cultures from male littermates. This phenomenon is not exclusive to the C57BL/6 genetic background and also was observed in cultures from FVB and C3H/HeJ mice, although sex had only a modest influence in cultures from BALB/c mice. The magnitude of the sexual dimorphism was determined by genetic background, confirming previous studies indicating that number of osteoblast progenitors and potential for osteoblastogenesis of bone marrow stromal cell cultures are influenced by the strain of inbred donor mouse [40]. Our findings are in accordance with previous results obtained in cells from Sprague-Dawley rats, suggesting that sex determines the potential for osteoblastogenesis in bone marrow stromal cells from rodents [41].

In humans and rodents, differences in the levels of androgens and estrogens between sexes influence skeletal function [4]. However, it is not likely that hormonal influences determined the sexually dimorphic potential for osteoblastogenesis observed, since cells were obtained from mice before they reached full sexual maturity. Therefore, genetic determinants may establish differences in osteoblastic cellular behavior, and this notion is supported by the observation that different genetic backgrounds exhibit varying degrees of sexual dimorphism in osteoblastic differentiation and function [5], [6].

Analysis of frequency of osteoblastic precursors and studies in bone marrow stromal cell cultures from *αSma-GFP* transgenic mice indicate that the sexually dimorphic potential for osteoblastogenesis is due to reduced number of mesenchymal cells in cultures from female mice. Suppressed proliferation in cells from female mice also contributes to this effect, although only cultures from C57BL/6 mice exhibit sex related differences in their capacity for cell proliferation, confirming the influence of genetic background on the extent of sexual dimorphism in osteoblastogenesis. Selected aspects of osteoblast differentiation are suppressed in osteoblast precursors from female *αSma-GFP* transgenic mice in comparison to cells from male littermates, and these differences might contribute to the effect of sex on the potential for osteoblastogenesis. No differences related to sex are observed in the activity of intracellular signaling pathways that regulate osteoblastogenesis, such as bone morphogenetic protein/signaling mothers against decapentaplegic, Wnt/β-catenin and insulin-like growth factor/Akt (data not shown) [42]. Mesenchymal stem cells harvested from human adipose tissue and murine skeletal muscle can differentiate into osteoblasts *in vitro* and in agreement with our observations, cells from female donors commit to the osteoblastic lineage to a lesser extent than cells from male individuals [43], [44]. Therefore, sexually dimorphic potential for osteoblastogenesis may be a characteristic shared by mesenchymal stem cells irrespective of species and tissue of origin.

In contrast to the observations in mature cultures of bone marrow stromal cells, only limited aspects of the osteoblastic phenotype displayed differences related to sex in primary calvarial osteoblasts. This discrepancy suggests that in male and female mice, factors determining osteoblastogenesis must act early during the differentiation program for sexual dimorphism to develop completely and compensating mechanisms might reduce the impact of sex on osteoblastogenesis as cells mature *in vivo*. An alternative explanation is the different developmental origin of the bones used to provide bone marrow stromal cells and osteoblasts, since long bones derive by condensation of mesenchymal cells in the limb bud, whereas parietal bones originate from the neural crest [45]. Studies investigating expression of sex steroid receptors in calvarial osteoblasts revealed that sex had only a modest effect on the expression of androgen and estrogen receptors (Table S4), suggesting that differential expression of sex steroid receptors does not mediate the effects of sex on osteoblast function.

In conclusion, sex determines cancellous bone architecture of C57BL/6 mice and sex genetic factors have an impact on osteoblastogenesis. Although sexual dimorphism in humans and rodents is pervasive, findings from studies limited to one sex often are generalized to both sexes, a practice that may lead to erroneous interpretation of experimental results [46], [47]. Sexual dimorphism extends to mechanisms regulating cellular function, either under physiological or pathological conditions, underlying the importance of declaring the donor sex for studies performed in cell cultures [48]. Our results confirm that sex influences the interpretation of studies addressing skeletal structure and function, as well as highlight the importance of preserving the identity of cell cultures from donors of different sexes when investigating the mechanisms that determine osteoblastogenesis [2].

## Supporting Information

Table S1 Forward (Fwd) and reverse (Rev) primers used for measuring mRNA levels of androgen receptor (*Ar*), estrogen receptor (*Esr*)*1* and *Esr2* by qRT-PCR. GenBank accession numbers indicate transcript variants with homologous sequences to primers. cDNA for construction of standard curves were obtained from Source BioScience (Nottingham, UK).
(DOCX)

Tabe S2 Relative expression of androgen receptor (*Ar*) and estrogen receptor (*Esr*)*1* and *Esr2* in bone marrow stromal cells from C57BL/6 littermate mice of both sexes, exposed for 7 days to 10 nM 1,25 dihydroxyvitamin D_3_ (Vit D_3_) or control vehicle (Veh).
(DOCX)

Table S3 Femoral length of male and female C57BL/6 mice was measured on images of dissected femurs fixed in 70% ethanol, obtained on a µCT 40 scanner (Scanco Medical AG, Bassersdorf, Switzerland).
(DOCX)

Table S4 Relative expression of androgen receptor (*Ar*), estrogen receptor (*Esr*)*1* and *Esr2* in calvarial osteoblasts from male and female FVB, C57BL/6, C3H/HeJ and BALB/c littermate mice.
(DOCX)
