# Supplementary material for: Sex and Genetic Factors Determine Osteoblastic Differentiation Potential of Murine Bone Marrow Stromal Cells
Source: PLoS One. 2014 Jan 28;9(1):e86757. doi: 10.1371/journal.pone.0086757 (PMC3904935; doi:10.1371/journal.pone.0086757)
Supplement: Table S1 — Forward (Fwd) and reverse (Rev) primers used for measuring mRNA levels of androgen receptor (Ar), estrogen receptor (Esr)1 and Esr2 by qRT-PCR. GenBank accession numbers indicate transcript variants with homologous sequences to primers. cDNA for construction of standard curves were obtained from Source BioScience (Nottingham, UK). (DOCX) [file pone.0086757.s001.docx]

**Table S1.**

| **Gene** | **Strand** | **Primer Sequence** | **GenBank Accession Numbers** |
| --- | --- | --- | --- |
| *Ar* | Fwd | 5’-GGACATGCGTTTGGACAG-3’ | NM_013476 |
|  | Rev | 5’-GGAAAGTAATAGTCGATGGGTAAA-3’ |  |
| *Esr1* | Fwd | 5’-CCTACTACCTGGAGAACGAG-3’ | NM_007956 |
|  | Rev | 5’-ATTGTCAGAATTAGACCTGTAGAAG-3’ |  |
| *Esr2* | Fwd | 5’-TCCACCCGCTAGGCATTC-3’ | NM_010157, NM_207707 |
|  | Rev | 5’-ACATGATGCTCTCAGAGACTCA-3’ |  |
