# Supplementary material for: Sex and Genetic Factors Determine Osteoblastic Differentiation Potential of Murine Bone Marrow Stromal Cells
Source: PLoS One. 2014 Jan 28;9(1):e86757. doi: 10.1371/journal.pone.0086757 (PMC3904935; doi:10.1371/journal.pone.0086757)
Supplement: Tabe S2 — Relative expression of androgen receptor (Ar) and estrogen receptor (Esr)1 and Esr2 in bone marrow stromal cells from C57BL/6 littermate mice of both sexes, exposed for 7 days to 10 nM 1,25 dihydroxyvitamin D3 (Vit D3) or control vehicle (Veh). (DOCX) [file pone.0086757.s002.docx]

**Table S2.**

|  | **Male** | | **Female** | | |  |
| --- | --- | --- | --- | --- | --- | --- |
|  | **Vehicle** | **Vit D_3_** | | **Vehicle** | **Vit D_3_** | |
| *Ar* relative expression | 1.00 ± 0.02 | 3.51 ± 0.71 | | 0.89 ± 0.06 | 4.09 ± 0.32 | |
| *Esr1* relative expression | Not detectable | | | | | |
| *Esr2* relative expression | Not detectable | | | | | |

Values are means ± SEM; n = 4. * Significantly between males and females, *p* < 0.05.
