# Supplementary material for: Sex and Genetic Factors Determine Osteoblastic Differentiation Potential of Murine Bone Marrow Stromal Cells
Source: PLoS One. 2014 Jan 28;9(1):e86757. doi: 10.1371/journal.pone.0086757 (PMC3904935; doi:10.1371/journal.pone.0086757)
Supplement: Table S3 — Femoral length of male and female C57BL/6 mice was measured on images of dissected femurs fixed in 70% ethanol, obtained on a µCT 40 scanner (Scanco Medical AG, Bassersdorf, Switzerland). (DOCX) [file pone.0086757.s003.docx]

| **Age (months)** | **Sex** | **Femoral length (mm)** | **p value, effect of sex** |
| --- | --- | --- | --- |
| 1 | Male | 11.86 ± 0.12 | 0.59 |
|  | Female | 11.76 ± 0.15 |  |
| 3 | Male | 15.35 ± 0.09 | 0.63 |
|  | Female | 15.28 ± 0.11 |  |
| 6 | Male | 15.82 ± 0.08 | 0.85 |
|  | Female | 15.78 ± 0.21 |  |

**Table S3.**

Values are means ± SEM, n = 24-38 mice.
