# Supplementary material for: Sex and Genetic Factors Determine Osteoblastic Differentiation Potential of Murine Bone Marrow Stromal Cells
Source: PLoS One. 2014 Jan 28;9(1):e86757. doi: 10.1371/journal.pone.0086757 (PMC3904935; doi:10.1371/journal.pone.0086757)
Supplement: Table S4 — Relative expression of androgen receptor (Ar), estrogen receptor (Esr)1 and Esr2 in calvarial osteoblasts from male and female FVB, C57BL/6, C3H/HeJ and BALB/c littermate mice. (DOCX) [file pone.0086757.s004.docx]

**Table S4.**

|  | **Confluence** | | **7 Days** | | |  |
| --- | --- | --- | --- | --- | --- | --- |
| **FVB** | **Male** | **Female** | | **Male** | **Female** | |
| *Ar* relative expression | 1.0 ± 0.1 | 1.1 ± 0.1 | | 2.2 ± 0.2 | 2.3 ± 0.1 | |
| *Esr1* relative expression | Not detectable | | | | | |
| *Esr2* relative expression | 1.0 ± 0.1 | 1.2 ± 0.2 | | 1.2 ± 0.2 | 1.1 ± 0.2 | |
|  | **Confluence** | | | **7 Days** | | |
| **C57BL/6** | **Male** | **Female** | | **Male** | **Female** | |
| *Ar* relative expression | 1.0 ± 0.1 | 1.3 ± 0.3 | | 3.6 ± 0.1 | 5.3 ± 0.2* | |
| *Esr1* relative expression | Not detectable | | | | | |
| *Esr2* relative expression | 1.0 ± 0.2 | 0.4 ± 0.1* | | 1.1 ± 0.1 | 0.8 ± 0.1* | |
|  | **Confluence** | | | **7 Days** | | |
| **C3H/HeJ** | **Male** | **Female** | | **Male** | **Female** | |
| *Ar* relative expression | 1.0 ± 0.1 | 1.1 ± 0.1 | | 14.5 ± 1.8 | 10.9 ± 1.4 | |
| *Esr1* relative expression | Not detectable | | | | | |
| *Esr2* relative expression | 1.0 ± 0.2 | 1.3 ± 0.1 | | 0.9 ± 0.1 | 1.5 ± 0.1* | |
|  | **Confluence** | | | **7 Days** | | |
| **BALB/c** | **Male** | **Female** | | **Male** | **Female** | |
| *Ar* relative expression | 1.0 ± 0.1 | 1.4 ± 0.1* | | 19.1 ± 3.0 | 13.5 ± 3.1 | |
| *Esr1* relative expression | Not detectable | | | | | |
| *Esr2* relative expression | 1.0 ± 0.2 | 1.3 ± 0.3 | | 0.6 ± 0.1 | 0.7 ± 0.1 | |

Values are means ± SEM; n = 4. * Significantly between males and females, *p* < 0.05.
